# Blood pressure changes during routine transthoracic echocardiography^☆^

**DOI:** 10.1016/j.ijcrp.2023.200170

**Published:** 2023-01-14

**Authors:** Mazhar Kadwalwala, Brian Downey, Ayan Patel, Monica Dehn, Benjamin Wessler

**Affiliations:** a Tufts Medical Center, 800 Washington Street, Boston, MA 02111, USA

**Keywords:** Echocardiogram, Blood pressure, Hypertension, Valvular disease, Afterload

## Abstract

**Background:**

Increased afterload affects many of the flow dependent metrics assessed during transthoracic echocardiography (TTE) especially in the evaluation valvular disease. A single timepoint blood pressure (BP) may not accurately reflect the afterload present at the time of flow-dependent imaging and quantification. We assessed the magnitude of change in BP at discrete timepoints during routine TTE.

**Method:**

We conducted a prospective study where participants underwent automated BP measurement while undergoing a clinically indicated TTE. The first reading was obtained right after the patient lay supine and subsequent readings were taken at 10-min intervals during image acquisition.

**Result:**

We included 50 participants (66% were male, with a mean age of 64 years). After 10 min, 40 (80%) participants had a drop in systolic BP of >10 mmHg. Compared to the baseline, there was a significant drop in systolic BP (mean decrease 20.0 ± 12.8 mmHg; P < 0.05), and diastolic BP (mean decrease 15.7 ± 13.2 mmHg; P < 0.05) at 10 min. The systolic BP remained different from the baseline value throughout the duration of the study (average decrease from baseline to study end was 12.4 ± 16.0 mmHg, p < 0.05).

**Conclusion:**

BP recorded just prior to TTE does not accurately reflect the afterload present during most of the study. This finding has important implications for valvular heart disease imaging protocols that incorporate flow dependent metrics, where the presence or absence of hypertension may lead to under- or over-estimation of disease severity.

## Highlights

• A single, initial BP is measured during routine transthoracic echocardiogram.
• However, SBP and DBP can drop by 20 and 15 mmHg respectively in first 10 minutes.
• A single initial measure may not reflect afterload during load-dependent imaging.
• This impacts the accuracy of flow-dependent valvular disease imaging protocols.

## 1 Introduction

Blood pressure (BP) influences several echocardiogram based structural assessments including ventricular size, function, and apparent severity of valve lesions [[1], [2], [3]]. Systemic hypertension is common for patients with complex imaging phenotypes including heart failure [4], and left sided valve diseases [5]. Numerous imaging guidelines incorporate the assessment of loading conditions to accurately grade specific lesions, with changes in loading conditions recognized as potential cause for misclassification of disease severity [6,7]. BP is dynamic and impacted by body positioning and activity. Standard outpatient guidelines recommend BP assessment after a patient is seated and relaxed with feet on the floor for greater than 5 min. Furthermore, to minimize random error 2–3 BP measurement should be obtained on 2–3 different occasions [8]. In standard outpatient echocardiography, initial BP measurement is taken when the patient is placed in the exam room, prior to image acquisition.

Current guidelines for conducting a comprehensive transthoracic echocardiogram recommend measurement and documentation of systemic blood pressure though do not offer any additional guidance [9]. As a result, it is likely that there is substantial variation in practice with respect to timing of afterload assessment and subsequent load-dependent imaging. We hypothesize that BP measured just prior to image acquisition may not reflect the afterload that is present during the time of load-dependent imaging.

## 2 Methods

This is a prospective study of serial BP measurements to assess for changes in loading conditions among patients referred for clinically indicated transthoracic echocardiogram (TTE) at a single academic medical center (Tufts Medical Center, Boston, USA). Participants were >18 years old and approached for consent once they were moved into a private examination room. Subjects gave written informed consent to participate in the study and the study was approved by the IRB at Tufts Medical Center as conforming to the ethical guidelines of the 1975 Declaration of Helsinki. Consistent with current guidelines [9], the protocol in the Tufts Cardiovascular Imaging and Hemodynamic Laboratory is to acquire BP, while patient is supine, at a single time point prior to TTE imaging. All blood pressure readings were collected using the same brachial cuff based automatic BP device (Philips SureSigns VM6) while patient lay supine. All readings were obtained in the presence of a physician and a sonographer. After initial vital signs were obtained, sonographers proceeded with standard TTE imaging protocols with standard patient positioning, and serial BP measurements assessed every 10 min until the study end.

### 2.1 Statistical analysis

BP and HR measurements were analyzed in 10-min intervals and at the end of the study (T_initial_, T_10_, T_final_). A change in BP and HR was calculated by finding the difference between systolic blood pressure (SBP) readings, diastolic blood pressure (DBP) readings, and HR readings taken at each discrete 10-min mark (T_10_-T_initial_, T_final_-T_10_). Since study times varied, after T_10_, a T_final_ time point was used. T_final_ reflects data recorded any time after and including 20 min from study start. SBP, DBP, and HR readings at the end of the study (T_final_) and at the start of the study (T_initial_) were also compared. A mean change was calculated between each interval. All calculated means were reported with their associated standard deviation (SD). Paired *t*-test was used to assess statistical significance.

A sub-group analysis to assess change in these vital signs was also done for participants based on whether they were undergoing treatment for hypertension at the time of the study. Welch's T-test was used to test for difference between the means measured.

## 3 Results

Baseline characteristics and mean SBP, DBP, and HR for T_initial_, T_10_ and T_final_ are shown in Table 1. 50 participants were enrolled in this study. 62% of participants were treated for hypertension at the time of their TTE. On average each study lasted for about 35.8 min (range 15–45 min).

**Table 1:** Summary of demographic data for patient population for the study as well as the mean values for multiple hemodynamic variables collected at discrete time points during the study.

Table 1: Demographic and hemodynamic data	
**Demographic Data (n** = **50)**	Mean (SD)
Age (years)	64.4 (16.6)
Gender (M: F)	33:17
Concurrent Hypertension Treatment (n)	31
**Body Mass Index (BMI)**	28.0 (5.4)
**Body Surface Area (BSA)**	1.9 (0.3)
**Hemodynamic variables**	
Heart rate (bpm) (n = 47)	
T_initial_	73.3 (17.3)
T_10_	70.9 (15.9)
T_final_	69.5 (13.9)
BP (mm Hg) (n = 50)	
T_initial_	134.2 (19.5)/78.7 (14.0)
T_10_	114.2 (17.6)/63.0 (14.8)
T_final_	121.8 (20.1)/71.9 (15.9)
LV Ejection Fraction (%) (n = 50)	57.9 (10.1)

**Fig. 1:**
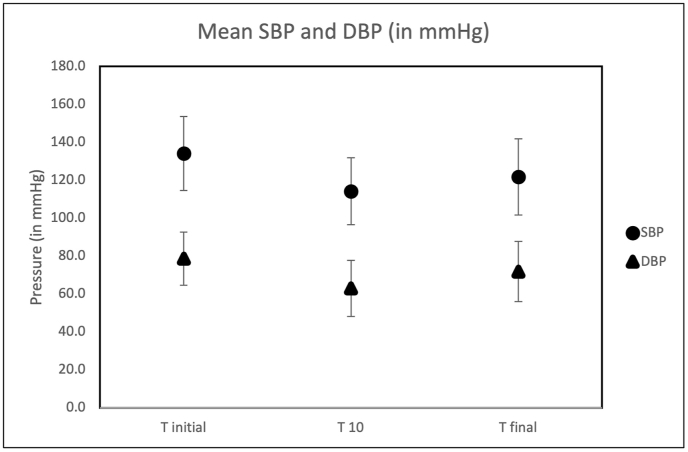
Mean systolic blood pressure (SBP) (●) and DBP (▲) and diastolic blood pressure (DBP) over the course of the TTE study. T_initial_ is the first BP reading and T_10_ represents the BP reading taken 10 min after the first reading. T_final_ is the BP reading taken at the end of the study.

In our study population, 22 (44%) participants were in the hypertensive range (stage I or higher) at T_initial_. At T_10_, only 5 (10%) participants were in the hypertensive range and at study end (T_final_) 11 (22%) participants were in the hypertensive range. The mean decrease in SBP from T_initial_ to T_10_ was 20.0 with an SD of 12.8 mmHg; (P < 0.05) and mean decrease in DBP was 15.7 with an SD of 13.2 mmHg; (P < 0.05). At T_10_, 40 (80%) participants had ≥10 mmHg decrease in SBP with 23 (46%) participants showing a ≥20 mmHg decrease in SBP. Similarly, with DBP, at T_10_, 33 (66%) had a ≥10 mmHg while 17 (34%) had a ≥20 mmHg drop in DBP. From T_10_ to T_final_ there was a mean increase in SBP of 7.6 with an SD of 13.0 mmHg; (P < 0.05) and in DBP of 8.9 with an SD of 12.8 mmHg; (P < 0.05). However, from T_initial_ to T_final_ there was a mean decrease in SBP of 12.4 with an SD of 16.0 mmHg (p < 0.05) and in DBP of 6.8 with an SD of 14.8 mmHg (p < 0.05). Fig. 1 shows the average change in hemodynamics over the course of the study. Heart rate (HR) did not change significantly throughout the study at any given discrete time interval.

A subgroup analysis was conducted by whether the participant was undergoing treatment with anti-hypertensive medications at the time of the TTE study. 31 participants were undergoing treatment for hypertension (HTN-Tx) at the time of the study and 19 participants were not (no HTN-Tx). The HTN-Tx group had statistically significant (p < 0.05) higher SBPs than the no HTN-Tx group at all reported time points. These findings are summarized in Table 2.

**Table 2:** Summary of the change in SBP and DBP from T_initial_ to T_10_ and T_10_ to T_final_ for each of the subgroups, with the corresponding p-values.

Table 2		Delta for HTN-Tx (in mmHg)	Delta for no HTN-Tx (in mmHg)	P-value
Average SBP	T_initial_ to T_10_	−20.9 ± 14.1	−18.6 ± 10.3	0.52
	T_10_ to T_final_	8.9 ± 10.6	5.5 ± 16.2	0.42
	T_initial_ to T_final_	−11.9 ± 14.8	−13.1 ± 18.2	0.81
Average DBP	T_initial_ to T_10_	−15.4 ± 13.7	−16.2 ± 12.6	0.84
	T_10_ to T_final_	7.2 ± 11.9	11.6 ± 14.2	0.26
	T_initial_ to T_final_	−8.2 ± 14.5	−4.6 ± 15.4	0.41

## 4 Discussion

The main finding from our study is that the office-based BP documented immediately prior to TTE imaging does not accurately reflect loading conditions present later in the imaging protocol. Our study showed a significant drop in blood pressure for 80% of the participants over the course of the study and 44% of the participants would have had blood pressure readings in the hypertensive range when they were in fact normotensive 10 min into the study. Blood pressure drop during the study is likely related to the prolonged period of supine rest that occurs during image acquisition.

Consideration could be given to changing the timing of BP measurement to 10 min after starting image acquisition to obtain a BP value that is more reflective of the loading conditions present during most of the study. Further, automatic blood pressure monitors can be used for certain cases (e.g. valve cases), so that BP measurements can be made throughout a study and recorded just prior to load-dependent imaging. The ease-of-use of these automatic devices make them minimally disruptive. Given the importance of accurately assessing afterload when evaluating several pathologic states, it appears appropriate to re-consider how BP is assessed, in a minimally disruptive manner, during routine TTE imaging.

### 4.1 Clinical implications

Our findings suggest that a single BP measurement recorded at the start of the study is insufficient and does not accurately predict the loading conditions present during much of the imaging protocol. BP is central to clinical decision-making and the presence or absence of hypertension can lead to misclassification of disease severity and downstream treatment recommendations [8]. In the case of valve diseases [9] patients are routinely asked to return for repeat imaging once BP is better controlled. Mischaracterization of BP can lead to under-treatment or delays in treatment of advanced valve disease and higher costs for patients and insurers. Some of this repeat testing (and treatment delays) can be avoided if BP is assessed closer to the time of load-dependent imaging.

The significance of our findings is further illustrated with two clinical cases. Consider a 75-year-old woman with dyspnea. She is referred for TTE to evaluate a systolic murmur where her initial blood pressure is documented at 162/84. Her imaging reveals aortic stenosis (AS) that appears severe based on the valve morphology (severely thickened with restricted motion) but the transvalvular hemodynamics fall into the moderate range. The stroke volume is low. In the setting of systemic hypertension, guidelines recommend treating her blood pressure and follow up imaging when she is normotensive. If in fact her blood pressure had normalized during the TTE (as we show frequently happens) her case would be labeled as low gradient severe AS and should be treated. These discrepant care pathways emerge from the observation that transvalvular flow is significantly impacted by afterload [10]. And the integrative approach to grading this valve lesion incorporates afterload.

These issues are also relevant for assessing mitral regurgitation (MR). There is extensive evidence demonstrating the apparent variability of MR with changes in blood pressure [[11], [12], [13], [14]]. In the setting of significant MR observed in the presence of high afterload, treatment of hypertension is often the first therapeutic target (prior to considering valve repair/replacement). When significant MR is seen in the setting of normal afterload the valve becomes the primary treatment target. Appropriate assessment and downstream decision-making for these cases requires accurate and timely assessment of afterload in order to reduce barriers and delays in care.

## 5 Conclusion

Current guidelines recommend the treatment of hypertension prior to assessing the severity of aortic and mitral valvular disease. Our study shows that during a routine TTE the SBP and DBP can drop as much as 20 mmHg and 15 mmHg respectively in the first 10 min. Therefore, BP recorded just prior to TTE does not accurately reflect the afterload present during most of the study. This finding has important implications for valvular heart disease imaging protocols that incorporate flow dependent metrics, where the presence or absence of hypertension may lead to under- or over-estimation of disease severity.

## References

[1] del Villar Candelas Pérez, Savvatis Konstantinos, López Begoña, Kasner Mario, Martinez-Legazpi Pablo, Yotti Raquel, González Arantxa, Díez Javier, Fernández-Avilés Francisco, Tschöpe Carsten, Bermejo Javier (2017). Impact of acute hypertension transients on diastolic function in patients with heart failure with preserved ejection fraction. Cardiovasc. Res..

[2] Akintunde A.A., Akinwusi P.O., Familoni O.B., Opadijo O.G. (2010). Effect of systemic hypertension on right ventricular morphology and function: an echocardiographic study. Cardiovasc J Afr.

[3] Hayek A., Derimay F., Green L., Rosset M., Thibault H., Rioufol G., Finet G. (2020). Impact of arterial blood pressure on ultrasound hemodynamic assessment of aortic valve stenosis severity. J. Am. Soc. Echocardiogr..

[4] Tsimploulis A., Lam P.H., Arundel C. (2018). Systolic blood pressure and outcomes in patients with heart failure with preserved ejection fraction [published correction appears in JAMA cardiol. 2018 apr 1;3(4):358]. JAMA Cardiol.

[5] Das P., Pocock C., Chambers J. (2000). The patient with a systolic murmur: severe aortic stenosis may be missed during cardiovascular examination. QJM.

[6] Zoghbi W.A., Adams D., Bonow R.O. (2017). Recommendations for noninvasive evaluation of native valvular regurgitation: a report from the American society of echocardiography developed in collaboration with the society for cardiovascular magnetic resonance. J. Am. Soc. Echocardiogr..

[7] Chair Baumgartner H., Co-Chair Hung J., Bermejo J. (2017). Recommendations on the echocardiographic assessment of aortic valve stenosis: a focused update from the European Association of Cardiovascular Imaging and the American Society of Echocardiography. Eur Heart J Cardiovasc Imaging.

[8] Whelton P.K., Carey R.M., Aronow W.S. (2017). Guideline for the prevention, detection, evaluation, and management of high blood pressure in adults: executive summary: a report of the American college of Cardiology/American heart association task force on clinical practice guidelines [published correction appears in hypertension. Hypertension.

[9] Mitchell C., Rahko P.S., Blauwet L.A. (2019). Guidelines for performing a comprehensive transthoracic echocardiographic examination in adults: recommendations from the American society of echocardiography. J. Am. Soc. Echocardiogr..

[10] Hayek A., Derimay F., Green L., Rosset M., Thibault H., Rioufol G., Finet G. (2020). Impact of arterial blood pressure on ultrasound hemodynamic assessment of aortic valve stenosis severity. J. Am. Soc. Echocardiogr..

[11] Sahn D.J. (1988). Instrumentation and physical factors related to visualization of stenotic and regurgitant jets by Doppler color flow mapping. J. Am. Coll. Cardiol..

[12] Grayburn P.A., Weissman N.J., Zamorano J.L. (2012). Quantitation of mitral regurgitation. Circulation.

[13] Irvine T., Li X.K., Sahn D.J., Kenny A. (2002).

[14] Kizilbash A.M., Willett D.L., Brickner M.E., Heinle S.K., Grayburn P.A. (1998). Effects of afterload reduction on vena contracta width in mitral regurgitation. J. Am. Coll. Cardiol..

